# Health-Related Quality of Life After Thyroid Cancer Surgery: A Single-Center, Cross-Sectional Study in Southern Vietnam

**DOI:** 10.7759/cureus.40496

**Published:** 2023-06-16

**Authors:** Tran Thanh Vy, Tran Le Thi Thanh Nam, Lam Thao Cuong, Ho Tat Bang

**Affiliations:** 1 Thoracic and Vascular Department, University Medical Center Ho Chi Minh City, University of Medicine and Pharmacy at Ho Chi Minh City, Ho Chi Minh, VNM; 2 Department of Cardiovascular and Thoracic Surgery, Faculty of Medicine, University of Medicine and Pharmacy at Ho Chi Minh City, Ho Chi Minh, VNM; 3 Faculty of Public Health, University of Medicine and Pharmacy at Ho Chi Minh City, Ho Chi Minh, VNM; 4 Department of Health Organization and Management, Faculty of Public Health, University of Medicine and Pharmacy at Ho Chi Minh City, Ho Chi Minh, VNM

**Keywords:** eortc qlq-c30, patient, surgery, quality of life, thyroid cancer

## Abstract

Background

Thyroid cancer is the most common malignant disease in the endocrine glands. Symptoms of the disease affect the functions of organs in the body. Although thyroid cancer is often considered a “good cancer” because it progresses slowly, the likelihood of successful treatment is quite high; what is special is that the effect on the quality of life (QoL) is on par with more severe types of cancer. Currently, studies on QoL assessment in thyroid cancer patients are quite limited in southern Vietnam. The present study investigated the potential risk factors of deterioration in QoL scores in thyroid cancer patients after thyroidectomy.

Methodology

A descriptive, cross-sectional study was performed on a total of 162 patients who were diagnosed with thyroid cancer and underwent thyroidectomy at the University Medical Center Ho Chi Minh City, Vietnam, from February to May 2023. Data were collected through face-to-face interviews with patients and from medical records. The European Organization for Research and Treatment of Cancer Quality of Life Questionnaire Core 30 was used to assess the QoL one month after thyroidectomy. Multivariable logistic regression was used to identify factors related to QoL with statistical significance set at p-value <0.05.

Results

The mean overall QoL in thyroid cancer survivors was 84.4 ± 10.00 (on a scale of 0-100, where 100 was the best). The results of multivariate logistic regression analysis showed that the factors related to QoL after thyroidectomy were surgery type (p < 0.001), having a comorbidity (p = 0.029), economic status (p = 0.026), and hormone disorder (p = 0.009).

Conclusions

Our study indicated that surgery type, having a comorbidity, economic status, and hormone disorders were independent risk factors for decreased QoL one month after thyroidectomy. It is necessary to thoroughly assess the QoL before and after surgery in thyroid cancer patients. Longer follow-up QoL studies should be performed with larger sample sizes for more accurate results.

## Introduction

The thyroid gland, consisting of two connected lobes, is one of the largest endocrine glands in the human body weighing 20-30 g in adults. Thyroid lesions are often found on the gland, with a prevalence of 4%-7%. Most of them are asymptomatic, and thyroid hormone secretion is normal [1]. Thyroid cancer is a type of cancer that begins in the thyroid gland, wherein cells grow out of control. Thyroid cancer affects the function of other organs due to disorders and excess or deficiency of hormones, causing decreased metabolism, heart rate, blood pressure, and body temperature, among others. Thyroid cancer is often called “good cancer” because of its slow progression and high likelihood of successful treatment [2]. However, thyroid cancer affects the quality of life (QoL) as much as more aggressive cancers [3].

In Vietnam, thyroid cancer ranks 10th in total cancer cases in both sexes and tends to increase rapidly [4]. Thyroid cancer is one of the five most common cancers in two big cities in Vietnam, Ho Chi Minh City and Hanoi. It is predicted that by 2025, in women, thyroid cancer will rank second after breast cancer in Vietnam [5].

Many methods exist to treat thyroid cancer such as surgery, chemotherapy, radiation therapy, radioactive iodine, targeted therapy, and hormone replacement [6]. Thyroidectomy is considered the golden therapy in the treatment of thyroid cancer. However, complications after thyroid cancer surgery significantly affect the QoL of patients. Previous studies have reported that thyroid cancer survivors have impairments in their QoL [2,7-10].

Currently, there are no studies on the QoL of thyroid cancer patients after surgery in Vietnam. Therefore, we performed this study to evaluate and identify the risk factors for decreased QoL in thyroid cancer patients undergoing thyroidectomy. This study can assist healthcare workers in selecting and designing appropriate interventions and patient care plans.

## Materials and methods

Study settings and design

A descriptive, cross-sectional study was conducted among thyroid cancer patients after thyroidectomy at the University Medical Center Ho Chi Minh City, southern Vietnam from February to May 2023. The study protocol and ethics were approved by the medical ethics committee of the University of Medicine and Pharmacy at Ho Chi Minh City (decision number: 22885/ĐHYD-HĐ). All procedures were performed according to the Declaration of Helsinki. Participants were provided with the study information and voluntarily participated after signing a consent form to participate. The information collected from patients was kept confidential and used for study purposes only. Whether they agreed to participate in the study did not affect their treatment at the hospital. This study has been reported in line with the strengthening the reporting of cohort, cross-sectional and case-control studies in surgery (STROCSS) criteria.

Study participants, sample size, and sampling

The inclusion criteria were as follows: age ≥18 years at diagnosis, primary thyroid cancer diagnosis with clinicopathological information, and undergoing thyroidectomy ≥1 month. The exclusion criteria were as follows: language barriers, severe cognitive impairment, mental diseases, long geographical distance from the study center, presence of other primary tumors, and refusal to participate.

The study used a formula to calculate sample size to estimate 1 mean with standard deviation σ = 6.3 (according to domestic research by Nhung et al. [11]) d = 1, Z(1 -α/2) = 1.96. The sample size was calculated to be 153. However, we added a loss rate of 5%, thereby increasing the minimum sample size to 162 individuals.

During data collection, we recruited all thyroid cancer patients after thyroidectomy at the University Medicine Center Ho Chi Minh City from February to May 2023. The study sample conveniently selected all patients who met the sampling criteria during the study period.

Data collection and tools

Face-to-face semi-structured interviews were offered to coincide with a future hospital appointment. QoL was assessed using the European Organization for Research and Treatment of Cancer Quality of Life Questionnaire Core 30 (EORTC QLQ-C30) [12]. The QLQ‐C30 includes 30 items and four main areas: global health status, financial difficulties, symptom scales, and functional scales. In addition, it includes five functional scales (i.e., physical, role, emotional, cognitive, and social functioning) and eight symptom scales (i.e., fatigue, nausea and vomiting, pain, dyspnea, insomnia, appetite loss, constipation, and diarrhea).

We investigated the effects of age (<55, ≥55 years), gender, education, marital status, economic status, area of residence, use of insurance, support from relatives, ability to pay for treatment, and understanding of treatment. We also collected the clinical and laboratory characteristics of patients, including body mass index (BMI), histology (papillary, follicular, medullary, and undifferentiated), clinical stage, surgery type, lymph node dissection, having a comorbidity, parathyroid function, thyroid function, and laryngeal nerve injury.

We collected variables by interviewing patients face-to-face and gathering information from their medical records and laboratory results.

Statistical analysis

The research data were entered using Epidata 3.1 software and analyzed using Stata 16.0 (StataCorp., College Station, TX, USA). The unpaired t-test and analysis of variance (ANOVA) test were used to measure the relationship with significance set at p-values <0.05. The Mann-Whitney test was used as an alternative to the t-test when the QoL scores are non-normally distributed, and the Kruksal-Wallis test was used if the ANOVA test was inappropriate. Multivariable linear regression was performed to determine the true association with QoL scores by including the factors considered in univariate analysis. Significance was set at p-values <0.05 in the multivariable linear regression model.

## Results

During the data collection process, we recorded nearly 190 accessible subjects, of whom eight refused to participate in the study. A total of 182 people agreed to be interviewed, and 20 patients participated in the pilot study to refine the questionnaire for linguistic compatibility. The remaining 162 people were included in the final analysis.

Characteristics of study participants

The general demographic information is shown in Table 1. The patients in our study had a mean age of 45.32 ± 12.96 years, with the youngest being 19 years old and the oldest being 78 years old. Overall, the majority of the participants were female (79.6%), were aged <55 years (74.7%), nearly 75% lived in other provinces and cities, 44.5% had high educational attainment, and 71.6% were married. In addition, most participants were living with other people, with living with children and spouses accounting for the highest proportion (77.6% and 69.2%, respectively). Nearly 75% of patients were economically dependent on family and society.

**Table 1:** Characteristics of socioeconomic information (n = 162). *: mean ± standard deviation; **: minimum-maximum

Characteristic	Frequency	Ratio (%)
Age (years)	45.32 ± 12.96*	(19–78)**
Age group (years)		
<55 years old	121	74.7
55 years old and over	41	25.3
Sex		
Female	129	79.6
Male	33	20.4
Accommodation		
Other provinces/cities	124	76.5
Ho Chi Minh City	38	23.5
Job		
Employees	55	34
Worker	20	7.4
Farmer	20	12.4
Trade	27	16.6
Freelance labor	4	2.5
Housewife	16	9.9
Retirement	26	16
Student	2	1.2
Other	0	0
Academic level		
≤Level 1	13	8
Level 2	41	25.3
Level 3	36	22.2
>Level 3	72	44.5
Marital status		
Single	18	11.1
Married	116	71.6
Widow/Separation, divorce	28	17.3
Cohabitation status		
Yes	156	96.3
No	6	7.3
Subjects cohabiting (n = 156)		
Child	121	77.6
Grandchildren	27	17.3
Older brother or sister or brother	14	9
Wife or husband	110	70.5
Parents	27	17.3
Other	1	0.6
Economic status		
Depends on family/society	43	26.5
Self-control	119	73.5

Characteristics of the treatment process

Table 2 shows that the majority of study participants received treatment support (91.4%), 14.2% thought they could not afford to pay for medical treatment, and 74.1% used health insurance, with 97.5% having knowledge about treatment.

**Table 2:** Characteristics of information about the treatment process (n= 162).

Characteristic	Frequency	Ratio (%)
Treatment support person		
Yes	148	91.4
No	14	8.6
Subjects supporting treatment (n = 148)		
Couple	83	56.8
Children	64	43.2
Parents	36	24.3
Siblings	27	18.2
Other	0	0
Ability to pay		
Yes	139	85.8
No	23	14.2
Insurance use		
Yes	120	74.1
No	42	25.9
Understanding treatment information		
Yes	158	97.5
No	4	2.5
Information source (n = 158)		
Healthcare staff	156	98.7
Internet	103	65.2
Those around	87	55.1
Books/newspapers/radio/TV	68	43
Other	3	1.9

Characteristics of the clinical and laboratory findings

As shown in Table 3, approximately 53% of patients had a normal BMI, all patients had papillary thyroid carcinoma, 82.7% had clinical stage I disease, 58.6% underwent thyroid lobectomy, 68.5% did not undergo lymph node dissection, and 68.5% had comorbidities.

**Table 3:** Clinical and paraclinical characteristics after surgery (n = 162).

Characteristic	Frequency	Ratio (%)
Body mass index		
Skinny (<18.5 kg/m^2^)	5	3.1
Normal (18.5–23 kg/m^2^)	86	53.1
Overweight – obesity (≥23 kg/m^2^)	71	43.8
Cancer stage		
Stage I	134	82.7
Stage II	26	16.1
Stage III	2	1.2
Stage IV	0	0
Histopathology		
Papillary	162	100
Follicular, medullary, undifferentiated	0	0
Surgical method		
Thyroid lobectomy	95	58.6
Total thyroidectomy	67	41.4
Lymph node dissection		
No	111	68.5
Yes	51	31.5
Comorbidities		
No	111	68.5
Yes	51	31.5
Comorbidities (n = 51)		
Hypertension	33	64.7
Diabetes	17	33.3
Cardiovascular desease	4	7.8
Kidney disease	1	2
Liver disease	3	5.9
Stomach disease	4	7.8
Musculoskeletal	0	0
Other	5	9.8
Difficulty breathing in the larynx		
No	162	100
Hoarseness		
No	160	98.8
Yes	2	1.2
Endoscopy – vocal cords with limited mobility		
No	0	0
One side	0	0
Both sides	0	0
Endoscopy – fixed vocal cords		
No	0	0
One side	0	0
Both sides	0	0
Feeling numb, shrinking limbs		
No	121	74.7
Yes	41	25.3
Total or ionic calcium concentration		
Normal	124	76.5
Increase	2	1.2
Reduce	36	22.2
Parathyroid hormone concentration		
Normal	146	90.1
Increase	2	1.2
Reduce	14	8.7
Symptoms of thyrotoxicosis		
No	155	95.7
Yes	7	4.3
Symptoms of hypothyroidism		
No	143	788.3
Yes	19	11.7
FT4 test results		
Normal	118	72.8
Increase	2	1.9
Reduce	41	25.3
Thyroid-stimulating hormone test results		
Normal	111	68.5
Increase	46	28.4
Reduce	5	3.1
Bleeding after surgery		
No	161	99.4
Yes	1	0.6
Surgical wound infection		
No	159	98.2
Yes	3	1.8
Scar length	96.4 ± 6.13	

Besides, among 162 patients who received surgery for thyroid cancer, the number of patients with symptoms of numbness and limb contracture accounted for nearly one-fourth. The results of the calcium and parathyroid hormone tests in the study participants were mostly normal, followed by a decrease in both 22.2% and 8.7%, respectively.

For thyroid function after surgery, it was found that 4.3% and 11.7% of patients had symptoms of thyrotoxicosis and hypothyroidism, respectively. Furthermore, thyroid function test results including free thyroxine decreased by 25.3% and increased by 1.3%. Free triiodothyronine was not surveyed. Thyroid-stimulating hormone increase accounted for 28.4% and the decrease accounted for 3.1%.

In addition, postoperative bleeding was seen in only one out of 162 patients. Infection was seen in three patients, and the surgical scar had an average length of 96.4 ± 6.13 mm.

QoL of patients with thyroid cancer

The mean overall QoL of thyroid cancer survivors was 84.4 ± 10.00, the score in general health status was 75.3 ± 14.0, financial difficulties was 33.3 (0-33.3), the functional scale was 84.6 ± 10.3, and the symptom scale was 13.9 (5.5-25). Symptoms of fatigue, insomnia, and pain were the most common and the most severe (Table 4).

**Table 4:** Quality of life (QoL) of patients with thyroid cancer.

Field	QoL score	Minimum	Maximum
General health	75.3 ± 14.0	33.3	100
Function	84.6 ± 10.3	53.3	100
Physical function	100 (93.3–100)	53.3	100
Operation function	100 (83.3–100)	33.3	100
Emotional function	74.2 ± 17.5	25	100
Cognitive function	83.3 (83.3–100)	33.3	100
Social function	66.7 (66.7–83.3)	33.3	100
Symptom	13.9 (5.5–25)	0	44.4
Symptoms of fatigue	22.2 (11.1–33.3)	0	66.7
Symptoms of pain	16.7 (0–16.7)	0	66.7
Symptoms of rapid breathing	0 (0–33.3)	0	66.7
Symptoms of Insomnia	33.3 (0–33.3)	0	100
Symptoms of anorexia	0 (0–33.3)	0	66.7
Symptoms of constipation	0 (0–33.3)	0	66.7
Diarrhea symptoms	0 (0–0)	0	66.7
Symptoms of vomiting/nausea	0 (0–0)	0	33.3
Finance	33.3 (0–33.3)	0	66.7
Summary score of QoL	84.4 ± 10.0	58.5	100
Mean ± standard deviation	Median (interquartile range)		

Factors associated with QoL in thyroid cancer patients

The results of the multivariate regression analysis (Table 5) showed that the factors related to the QoL of thyroid cancer patients after surgery included surgery type (p < 0.001), having a comorbidity (p = 0.029), economic status (p = 0.026), and hormone disorder (p = 0.009). Further analysis showed that total thyroidectomy, having comorbidity, economic status depending on family and society, and increased FT4 were related to the QoL score.

**Table 5:** Results of the composite multivariate regression model. EORTC QLQ-C30: European Organization for Research and Treatment of Cancer Quality of Life Questionnaire Core 30

EORTC QLQ-C30	General health		Function	
	Coefficient	95% CI	Coefficient	95% CI
Economic status (dependent)	-7.3	-13.7 to -0.9		
Type surgery (total thyroidectomy)				
	-12.6	-18.7 to -6.4	-5.4	-10.2 to -0.6
Comorbidities (present)			-4.5	-8.2 to -0.7
FT4 concentration				
Normal			1	
Increase			-18.3	-31.9 to -4.7
Reduce			-4.9	-12.3 to 2.5

## Discussion

This study aimed to evaluate the health-related quality of life (HRQoL) of thyroid cancer after surgery in southern Vietnam and to explore the important correlates defining HRQoL. The results of our study indicated decreased QoL in thyroid cancer survivors. The surgery type, comorbidity, economic status, and hormone disorders were independent risk factors for decreased QoL one month after thyroidectomy.

The study was conducted among thyroid cancer patients who were operated on at the University Medicine Center in Ho Chi Minh City. The average age of the study participants was 45.32 ± 12.96 years, with the youngest being 19 years old, and the oldest being 78 years old. This result is similar to the study of Nhung et al. who reported an average age of 43.65 ± 12.09 years [11]. The ratio between the two groups under 55 years old and over 55 years old in our study was 3:1. However, in other studies on thyroid cancer, the proportion of age groups over 50 years accounted for a higher proportion [7]. In our study, we found that thyroid cancer was found mostly in women, accounting for 79.6%. This ratio is quite similar to the data reported by GLOBOCAN 2020 where the female/male ratio worldwide and in Vietnam was 3:1 and 4:1, respectively. The study by Nguyen et al. carried out in Vietnam also predicted that thyroid cancer will be the most common cancer in women after breast cancer in 2025 [5]. Other studies on thyroid cancer globally and in Vietnam have also shown that the majority of patients are women [7-11]. The sex hormone estrogen stimulates the proliferation of thyroid cancer cells, which may contribute to thyroid cancer, as has been shown in several studies globally [13]. Most study participants had high education, with 44.5% having education above high school. This result is similar to another study conducted in Asia [11].

We measured the QoL score using the EORTC QLQ-C30 scale. The validity and reliability of the toolkit have been proven by many studies and shown to have high validity and reliability. The internal consistency of EORTC QLQ-C30 is measured by Cronbach’s α coefficient for each domain. The Cronbach’s α value higher than 0.7 is generally considered satisfactory [14]. Our results showed that Cronbach’s α coefficient for the whole questionnaire was 0.8327.

In the area of ​​general health self-assessed by patients, our study obtained a result of 75.3 points. Meanwhile, according to Nhung et al., the score was 69.6. Another study reported the QoL score in the field of general health to be 72.3. Although our study results are higher, there is not much difference.

The average functional score in our study was 84.6 points, wherein the social function area had the lowest QoL score, followed by the emotional function. The results are similar to the results of a study conducted in northern Vietnam [11].

Regarding emotional functioning, patients have fears or concerns about their future health and they feel that they may often be angry, anxious, and fearful [10]. Performing screening, counseling, and treatment of depression for patients with thyroid cancer is being directed by medical professionals [3]. Psychological problems caused by thyroid cancer can adversely affect the patient’s QoL, thereby also partly affecting work and life. The patient’s daily activities lead to a decrease in the social function index score and integration into the community.

The symptom that the patient complains about is the reason to bring the patient to the hospital for examination; moreover, this is something that the health workers care about and want to improve. In our study participants, common symptoms included insomnia, fatigue, and pain. This result is similar to other studies on the QoL of patients with thyroid cancer after surgery [7,11]. Some studies have suggested that the reduced QoL is due to fatigue related to short-term or long-term prostate cancer after treatment. In addition, patients claim that they suffer from frequent insomnia along with psychological stress related to this symptom [15].

The treatment of cancer is often prolonged and many times supportive treatments such as hormones and radiation therapy affect the finances and the patient’s ability to pay. Therefore, the financial score in our study was 33.3. The higher this score, the greater the financial hardship the patient faces.

The mean overall QoL score of patients after thyroid cancer surgery in this study was 84.4 ± 10.0. Similarly, the score reported in the study by Liu et al. (84.4 ± 12.7) was higher than that reported by Li et al. (65.93 ± 9.00) but lower than that reported by Nhung et al. (91.76 ± 6.30). These studies use the same scale on the same patient population as our study [7,11,16]. The subjects participating in our study all had surgery ≥1 month, so at this time, temporary postoperative complications such as laryngeal nerve damage, hypoparathyroidism, and underlying hypothyroidism were still present and significantly affected patients. In contrast, Li et al. recruited patients three months after surgery, and Nhung et al. after six months. Such different QoL scores are partly due to the different study locations, which leads to the difference in results between these studies.

Further analysis showed that total thyroidectomy, having comorbidity, economic status depending on family and society, and increased FT4 were related to the QoL score. These factors had similarities with many previous studies conducted among thyroid cancer patient populations.

The most commonly used surgical approach in patients with thyroid cancer involves total or lobectomy. Each method has a certain impact on the QoL of the patient. We conducted a survey and found that surgical methods affect four out of four areas in the QoL scale. In the same study by Wang et al., Li et al., and Nickel et al., the specific type of surgery, total thyroidectomy, was also one of the predictors of a reduction in the QoL score [2,7,17]. However, a study done in Saudi Arabia reported that the surgical method did not affect the QoL outcome of patients with thyroid cancer 5-15 years after surgery [10]. The results are different. It can be explained that the time of the post-surgery survey in patients with thyroid cancer in each study is different, so the effect on QoL is different.

We found an association between QoL and preoperative comorbidities. Most of our patients often suffer from hypertension and diabetes, which are chronic non-communicable diseases that are thought to be common worldwide. The impact of the disease on the patient’s QoL is immeasurable. When the chronic disease is long-term, accompanied by the deterioration of health when the patient has cancer, the patient will have certain feelings and complaints about their QoL. There are also similarities with other studies. Goldfarb et al. concluded that thyroid cancer patients with comorbidities had decreased QoL [8]. However, our study is different from other studies in Vietnam in this regard [11].

We found the influence of occupation and economic status for the majority of the QoL scores. Our study results are consistent with the study of Wang et al. (6.47) [2]. Self-reliant patients are often still able to work, and their occupation is also stable, which means their health is better, their function is better, and their symptoms are less severe. Hence, their financial situation will be less difficult than those who have to depend on the help of family and society.

FT4 and thyroid-stimulating hormone are considered as one the first tests to determine thyroid function in patients who have undergone thyroid surgery [18]. Li et al.suggested that thyroid function including FT4 and TSH should be checked to investigate the QoL in patients with thyroid cancer after surgery [7]. Unexpectedly, we found that postoperative thyroid function including symptoms, FT4, and thyroid-stimulating hormone levels had an effect on the QoL of patients. One possible explanation for the decrease in QoL could be that hormone therapy failed to normalize FT4 levels in patients. Thus, it prolongs symptoms of thyroid dysfunction and reduces the QoL in thyroid cancer patients.

This study had some limitations. The number of patients was small. This study only evaluated the patient’s QoL at a time. Therefore, building a study with a larger number of patients and monitoring and evaluating the QoL of patients before and after surgery will provide a more comprehensive and accurate view. In this study, other factors including disease stage, histopathology, age, and gender did not have an effect on the QoL of the study participants, which was different from other similar studies. Future studies should perform in-depth assessments of QoL before and after surgery in patients with thyroid cancer. QoL studies with long-term follow-ups are needed for more objective and accurate findings.

## Conclusions

This study indicates that people with thyroid cancer who have had surgery often have HRQoL similar to or slightly worse than the general population. Insomnia symptom index, followed by fatigue symptoms, affects the QoL score of patients with thyroid cancer after surgery. Independent risk factors that reduce the QoL in patients after surgery include the surgical method, economic status, comorbidities, thyroid hormone excess, or deficiency.

## Appendices

**Table 6:** Study questionnaire.

Questionnaire							
Voting code:..........							
Investigation date:							
	Question	Answer	Code		Note		
	Part A: Background information						
A1	Year of birth	…					
A2	Sex	Male	0				
		Female	1				
A3	Job	Employees	0				
		Worker	1				
		Farmer	2				
		Trade	3				
		Freelance labor	4				
		Housewife	5				
		Retirement	6				
		Unemployment	7				
		Student	8				
		Other: (specify)…					
A4	Accommodation	Ho Chi Minh City	0				
		Other Provinces/Cities	1				
		(specify).………………					
A5	Academic level?	≤Level 1	0				
		Level 2	1				
		Level 3	2				
		>Level 3	3				
A6	Current marital status?	Single	0				
		Living with a spouse	1				
		Separation, divorce	2				
		Widow	3				
A7	Do you currently live with anyone?	No	0		If you choose 0, go to sentence A9		
		Yes	1				
			No	Yes	Many choices		
A8	Mr./Ms live	Child	0	1			
	with whom?	Grandchildren	0	1			
		Brother or sister or brother	0	1			
		Wife or husband	0	1			
		Parents	0	1			
		Other (specify)……………					
A9	Your current economic status?	Self-control	0				
	how?	Depending on family	1				
		Depends on social assistance	2				
		Other (specify)…………	3				
A10	Does anyone support you during your treatment?	No	0		Select 0 switch to A12		
		Yes	1				
A11	Who is your support person during your treatment?		No	Yes	Many choices		
		Parents	0	1			
		Couple	0	1			
		Children	0	1			
		Siblings	0	1			
		Other	0	1			
		specify if “other” is selected....................					
A12	Can you afford to pay the cost of medical treatment?	No	0				
		Yes	1				
A13	Do you use health insurance during your treatment?	No	0				
		Yes	1				
A14	Do you know about treatment information?	No	0		Select 0 switch to A15		
		Yes	1				
			No	Yes	Many choices		
A15	Where did you get that information from?	Healthcare staff	0	1			
		Newspaper/radio/television	0	1			
		Internet	0	1			
		Those around	0	1			
		Other (specify) …………	0	1			
	Part B: Disease information						
	(look up medical records)						
B1	BMI	Height…cm					
		Weight… kg					
B2	Disease stage	Stage I	0				
		Stage II	1				
		Stage III	2				
		Stage IV	3				
B3	Histopathology	Papillary	0				
		Follicular	1				
		Medullary	2				
		Undifferentiated	3				
B4	Surgical method	Thyroid lobectomy	0				
		Total thyroidectomy	1				
B5	Lymph node dissection	No	0				
		Yes	1				
B6	Does the patient have comorbidities?	No	0		If select 0, switch to C1		
		Yes	1				
			No	Yes	Many choices		
B7	What is the patient’s comorbidities?	Hypertension	0	1			
		Diabetes	0	1			
		Heart	0	1			
		Kidney	0	1			
		Liver	0	1			
		Stomach	0	1			
		Musculoskeletal	0	1			
		Other: (specify) ………….					
	Part C: Clinical and paraclinical features after surgery						
	(Looking up medical records)						
C1	Laryngeal nerve damage	Difficulty breathing in the larynx	No	0			
			Yes	1			
			C2	Hoarseness			No	0			
Yes		1								
C3		Otolaryngoscopy – Vocal cords with limited mobility	No	0			
			One side	1			
			Both sides	2			
C4		Otolaryngoscopy – Fixed vocal cords	No	0			
			One side	1			
			Both sides	2			
C5		Hypoparathyroidism	Feeling numb, shrinking limbs	No	0			
	yes			1			
C6	Total or ionic calcium concentration (if any) .....		Normal	0			
			Increase	1			
			Reduce	2			
			C7	PTH concentration (if any)			Normal	0			
Increase	1									
Reduce	2									
C8	Thyroid function after surgery		Symptoms of thyrotoxicosis: shaking hands, palpitations, hot flashes, sweating, weight loss, etc.	No	0			
		Yes		1			
C9		Symptoms of hypothyroidism: lethargy, sluggishness, cold and dry skin, weight gain, slow heart rate, etc.	No	0			
			Yes	1			
C10		FT4 test results	Normal	0			
			Increase	1			
			Reduce	2			
C11		TSH test results	Normal	0			
			Increase	1			
			Reduce	2			
C12	Condition of old surgical scars	Bleeding after surgery	No	0			
			Yes	1			
C13		Surgical wound infection	No	0			
			Yes	1			
C14		Scar length					
		……mm					
Part D: Questions EORTC QLQ-C30							
	Question	Do not have	Little	Much	So many		
D1	Do you find it difficult to perform strenuous tasks?	1	2	3	4		
D2	Do you find it difficult to walk long distances indoors?	1	2	3	4		
D3	Do you find it difficult to walk a short distance outside?	1	2	3	4		
D4	Do you need to stay in bed all day?	1	2	3	4		
D5	Do you need help with eating, dressing, bathing, or cleaning?	1	2	3	4		
D6	Do you have restrictions on your work or daily routines?	1	2	3	4		
D7	Are you limited in your pursuit of hobbies or recreational activities?	1	2	3	4		
D8	Do you have rapid breathing?	1	2	3	4		
D9	Are you in pain?	1	2	3	4		
D10	Do you need a break?	1	2	3	4		
D11	Do you have insomnia?	1	2	3	4		
D12	Do you feel weak?	1	2	3	4		
D13	Do you have anorexia?	1	2	3	4		
D14	Do you feel nauseous?	1	2	3	4		
D15	Do you have vomiting?	1	2	3	4		
D16	Are you constipated?	1	2	3	4		
D17	Do you have diarrhea?	1	2	3	4		
D18	Are you tired?	1	2	3	4		
D19	Are you affected by pain?	1	2	3	4		
D20	Do you find it difficult to focus on work such as watching TV or reading newspapers?	1	2	3	4		
D21	Do you feel stressed?	1	2	3	4		
D22	Do you feel worried?	1	2	3	4		
D23	Do you feel irritated easily?	1	2	3	4		
D24	Do you feel bored?	1	2	3	4		
D25	Do you find it difficult to recall an incident?	1	2	3	4		
D26	Is your physical condition or treatment interfering with your family's life?	1	2	3	4		
D27	Does your physical condition or treatment interfere with your social functioning?	1	2	3	4		
D28	Is your physical condition or treatment causing financial hardship for you?	1	2	3	4		
D29: Mr. (Mrs.) self-assessed overall health score							
	1	2	3	4	5	6	7
Very poor						Great	
D30: Mr. (Mrs.) self-assessment of quality of life score							
	1	2	3	4	5	6	7
Very poor						Great	
Notes about paraclinical results							
Laryngeal nerve injury diagnosed according to ATA 2015							
- Clinical: hoarseness or laryngeal dyspnea							
- Otolaryngoscopy: vocal cords restricted to mobility (comparison with contralateral) or fixed (unilateral or bilateral)							
Hypoparathyroidism was diagnosed according to ATA 2018.							
- Clinical: numbness, paresthesia, spasticity, tetany, Chvostek's sign or Trousseau's sign, ...							
- Subclinical: PTH and total calcium.							
Thyroid function after surgery							
+ FT4 and TSH: Both indexes are graded: Decrease, normal, and increase according to the reference range. Assess thyroid function status according to ATA.							
Thyroid function	TSH	FT4					
Thyroid toxicity	Reduce	Increase					
Subclinical thyrotoxicosis	Reduce	Normal					
Hypothyroidism	Increase	Reduce					
Subclinical hypothyroidism	increase	Increase					
The test results were compared with the reference range of each type of testing machine at the hospital - where the study was performed.							
